# Accurate FPGA-Based Velocity Measurement with an Incremental Encoder by a Fast Generalized Divisionless MT-Type Algorithm

**DOI:** 10.3390/s18103250

**Published:** 2018-09-27

**Authors:** Aleš Hace, Milan Čurkovič

**Affiliations:** Institute of Robotics, Faculty of Electrical Engineering and Computer Science, University of Maribor, Koroška cesta 46, SI-2000 Maribor, Slovenia; milan.curkovic@um.si

**Keywords:** incremental encoders, velocity measurement, MT method, FPGA, motion control

## Abstract

Velocity measurement by an incremental encoder is an important issue for advanced motion control applications such as robotics. In this paper, we deal with a kind of MT-type velocity estimation method. Though the conventional MT method is well known and has been well proven in practice, it requires execution of an arithmetic division operation that prevents an efficient implementation on low-cost FPGA-based control platforms. Thus, we propose a divisionless MT-type algorithm, which can provide a similar performance in velocity estimation accuracy as the conventional method, but requiring significantly less FPGA resources, since it implements only simple arithmetic operations such as addition, subtraction, and multiplication, that can be implemented more easily on the processing hardware. Furthermore, the algorithm is fast in execution, thus, it provides the output in only a few clock cycles. Though the proposed algorithm can be described in a recursive form, the stability of the estimation process is not jeopardized, although it is an important issue in this case. Hence, the algorithm is introduced in a form which assures stability in a wide speed range. We show the implementation of the algorithm on the experimental FPGA platform. The experimental results validated the proposed divisionless MT-type algorithm fully for accurate velocity estimation.

## 1. Introduction

Advanced motion control applications require highly accurate wide-range velocity information with high-bandwidth [1,2,3,4]. A typical motion control system consists of a motorized drive, a feedback device and a controller. The latter should generate the necessary control decisions in order to achieve the demanded system performance; however, to be able to do so, it requires proper feedback information. In motion control applications the system stability is of primary concern. Though there are numerous control architectures in use, the velocity feedback plays a crucial role in assuring the stability of the system and in the disturbance rejection. Velocity estimation is also essential for precision and performance of the motion control, which requires high-accuracy and high bandwidth velocity feedback information. Typical feedback devices in such systems are incremental position encoders, which have been used widely for position measurement in digital control systems of servo drives for several decades [5]. Usually, the feedback signal for the velocity controller is calculated from the measured position obtained by the incremental encoder [6]. However, due to the discrete nature of the incremental encoder operating as a pulse generator, a straightforward approach for velocity estimation results in highly noisy data that cannot usually be applied directly in the control loop. Though the noise component can be filtered out, this approach causes a phase lag that is undesired in high-performance closed-loop control in the applications of robotics or haptic interfaces [3,4,7].

There has been extensive research over the past few decades in order to provide accurate and delay-less smooth velocity information over a wide speed range from a digital incremental encoder. An excellent survey is provided in [8,9]. The simplest velocity estimation approach is based on counting encoder position pulses within a sampling period, as is the case in the well-known M-method. However, the M-method produces highly noisy output, especially at high sampling rates, due to the spatial position quantization inherent to incremental encoders. The alternative T-method can be utilized instead, in which the time interval between two adjacent encoder pulses should be measured by counting high-frequency clock pulses. The velocity information is then obtained by the reciprocal of the measured time interval, i.e., by arithmetic division. Though the method can provide fine velocity estimation at low speed, it is prone to errors at high speed. Thus, the main stream of the velocity measurement methods combine the M-method and the T-method. Since the pioneering work by Ohmae [10], the MT-method has been applied widely, because it works well in wide speed ranges and also has a high accuracy in the low speed range. Some variations of the MT-method appeared in [11,12,13]. They can be classified as the MT-type methods which may provide highly smooth velocity with no phase lag in a wide speed range. Further research deals with performance improvement of the MT-method and its enhanced robustness to the hardware inaccuracies [2,14,15,16]. Time stamping of encoder pulses presents a generalization of the MT method [17]. Here, the velocity is estimated by a polynomial fitting through a number of time-stamped encoder counts, which increases computation complexity significantly. On the other hand, Zhu [18] proposed an MT-type method with an efficient processing algorithm in order to simplify the required real time computation effort.

The MT-method is a high-performance velocity estimation approach that requires dedicated hardware, which should provide counting encoder pulses and measurement of elapsed time intervals related to the encoder pulses. It can be incorporated easily into digital processing systems with a fixed sample period. However, processing requires execution of some arithmetic operations, including multiplication and division. The latter is a much more demanding operation for processing hardware than multiplication [19]. Though advanced DSP processors allow implementation with the arithmetic division with no significant effort [20], the system design complexity may increase significantly in the case of advanced motion control applications with high sampling rates, which require special hardware interfaces usually unavailable in commercial DSP controllers. On the other hand, digital programmable circuits such as FPGAs, which are, nowadays, used widely for many industrial applications [21,22], offer possibilities for integrating complex hardware systems and their interfaces into a single FPGA chip. FPGA devices have reached a high level of development that puts them in competition with the traditional Application Specific Integrated Circuits (ASICs) in terms of performance, power consumption and cost, which make them most suitable for smart, intelligent and reconfigurable low-power sensor systems in different application fields [23,24,25,26]. Thus, they can also easily be designed for custom conditioning circuits required for improved velocity estimation methods [27] in advanced motion control applications that require not only highly accurate and short sampling periods, but also a short processing time of control algorithms. Furthermore, the low cost of recent FPGAs and the capacity of these devices enable economical implementation of a custom complex circuitry featuring parallel operation and low power consumption in a small space. This allows development of new high-performance applications; however, complex computation algorithms still present a design challenge [28].

In our research, we are concerned with the implementation of the MT-method on an FPGA. Our focus is on the data processing part that involves a computation with an arithmetic division, which is particularly difficult for FPGA-based embedded electronics to perform [29,30], and is characterized with high computation latency and high consumption of FPGA resources [31]. Therefore, for efficient implementation of the MT-method on an FPGA, it is necessary to circumvent the division operation inherent in the conventional algorithm. On the other hand, multiplication can be implemented easily in an efficient way, since some FPGAs embed dedicated multipliers, thus eliminating the need for using FPGA distributed logic cells [32]. Zhu [18] proposed a solution which combines the M-method and the T-method seamlessly, in which the arithmetic division is performed by the sophisticated mechanism with two counters and an accumulator. However, despite the simplicity of the method, it requires execution of the algorithm continuously in every clock cycle that increases resources and power consumption of the digital circuit, which is undesired. Especially, the latter has become a first order concern for VLSI design engineers [30]. Hace [33] introduced a novel MT-type method, which eliminates the division from the algorithm. Hence, it is called a DivisionLess MT method (DLMT). It can be performed by simpler arithmetic operations, such as addition and multiplication, yet produces an output very close to the conventional MT-method.

In this paper, we redesign the DLMT method algorithm, which is described in a recursive form. Due to the recursive nature of the algorithm, stability is an issue and, thus, it has already been investigated in [33]. Asymptotic stability has been shown clearly; however, the case of blank intervals (sampling intervals with no encoder pulses’ occurrence), which may appear sporadically or as a series of contiguous blank sampling intervals, has been not covered. As is explained later in Section 2 of the current paper, the blank intervals can cause instability of the recursive DLMT algorithm. Therefore, we modified it and thus now we introduce the Generalized DivisionLess MT algorithm (GDLMT) that assures asymptotic stability in all speed ranges, including the case when encoder pulses are widespread. The proposed algorithm, which has been designed for efficient FPGA implementation while preserving utmost accuracy of the conventional MT-method, presents the main contribution of the paper. It has been implemented on the FPGA-based platform and validated experimentally with an industrial optical rotary incremental encoder. The experimental results show excellent matching with the conventional MT-method. Thus, the latter can be replaced successfully by the proposed GDLMT algorithm, which provides an efficient FPGA implementation, since it requires significantly less resources and provides fast execution that is highly desirable in advanced motion control applications with short sampling periods.

## 2. Materials and Methods

### 2.1. Basics of Incremental Encoders

Incremental encoders are used mainly for position measurement by counting the digital pulses which they produce. Without loss of generality, we can assume a rotary incremental encoder in our study. The basic principle of its operation can be described such that it transforms mechanical motion into electrical output, thus providing digital pulses while its shaft is rotating, allowing measurement of the relative displacement of the shaft [34]. The number of generated output pulses is proportional to its relative angular position of the shaft, while the frequency of them is proportional to the angular velocity. In a single turn, the encoder generates a certain number of pulses, such that the angular step graduation given by the encoder pitch Θ*_p_* can be calculated as:

(1) $$\Theta_{p}=(2\pi )/N,$$

where *N* stands for the number of pulses generated by the encoder per one revolution of the shaft. In our study, we consider quadrature incremental encoders that output two channels of pulses, i.e., A and B channels, with the same number of digital pulses generated. However, the pulses in channel B should be shifted in phase by 90 degrees w.r.t. the pulses in channel A. The phase shift between the A and B channel pulses enables us to discriminate the direction of movement. In order to accurately read the pulses, which are generated by the incremental encoder, and to measure the relative angular position of the shaft, it is necessary to connect the encoder electrical lines to a dedicated hardware electronic circuit that provides proper conditioning of the digital signals, pulses decoding and bidirectional counting [27]. Such hardware component is usually called a quadrature decoder [35]. A quadrature decoder is applied for decoding the output of a quadrature encoder and, thus, it counts the pulses, incrementing or decrementing, as determined by the decoded motion direction. An enhanced quadrature decoder can be supplemented in such a way that it also provides measurement of the elapsed time interval since the recent encoder pulse was acquired.

### 2.2. The Simple M-Method

The simplest way to measure velocity by an incremental encoder is to count encoder pulses generated within a single sampling time interval. If we assume that the position can be obtained by accumulating the counted pulses, then the velocity is obtained as:

(2) $$v_{k}^{M}=\frac{x_{k}-x_{k-1}}{T_{s}},$$

where $x_{k}$, and $v_{k}^{M}$ are the acquired encoder positions sampled in the *k*-th sampling instant, and the corresponding velocity estimation, respectively. $T_{s}$ is the sampling time period. This method works as a frequency counter, producing an output alternating between a discrete number of the counted encoder pulses, which occur asynchronously with the sampling instants. Thus, especially in the low-speed region where only a small number of pulses are generated, the method produces considerable noise in the output due to the spatial quantization given by the encoder pitch (1). However, one can note that the division with the constant sampling period $T_{s}$ can be replaced by multiplication with the constant sampling frequency $F_{s}=1/T_{s}$. Thus, the M-method is rather simple in terms of computation complexity.

### 2.3. The Conventional MT-Method

**Figure 1:**
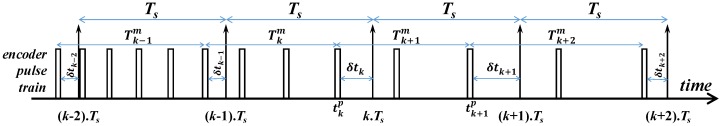
The timing diagram of encoder pulse train with sampling periods.

The conventional MT-method for velocity measurement can be described as [8]:

(3) $$v_{k}^{MT}=\frac{x_{k}-x_{k-1}}{T_{s}+\delta t_{k-1}-\delta t_{k}},$$

where $x_{k}$, $\delta t_{k}$ and $v_{k}^{MT}$ are the acquired encoder positions, the elapsed time interval since the recent encoder pulse (sampled in the *k*-th sampling instant), and the estimated velocity calculated by the formula, respectively (please see Figure 1 for details).

Though the sampling period is normally fixed, the measurement time interval $T^{m}$, which extends between the edges of the first and the last position pulse captured in the measurement window, varies accordingly. Thus, smooth velocity output can be obtained that actually represents accurate average velocity on the measurement interval. However, at low velocity, it may happen that encoder pulses may not occur in every sampling interval, but a single blank sampled period or a series of blank sampling periods may appear before the next encoder pulse occurrence. Then, the previous formula can be rewritten as:

(4) $$v_{k_{i}}^{MT}=\frac{x_{k_{i}}-x_{k_{i}-m_{i-1}}}{m_{i-1}T_{s}+\delta t_{k_{i}-m_{i-1}}-\delta t_{k_{i}}},$$

where $m_{i}=1+n_{i}$, and $n_{i}$ determines the number of blank sampling intervals. It means that if some encoder pulses appear in the $k_{i}$-th sampling interval, then this is followed by $n_{i}$ blank sampling intervals, such that:

(5) $$k_{i+1}=k_{i}+n_{i}+1,$$

**Figure 2:**
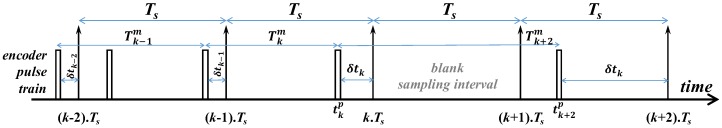
The timing diagram of encoder pulses widespread with a blank sampling interval.

Thus, between the sampling intervals with encoder pulse occurrence there are *n_i_* blank sampling intervals. The measurement interval is then extended for the same number of intervals. Note that the velocity estimation formula is calculated at the non-blank sampling intervals only. The timing diagram with rare encoder pulses and a blank sampling interval are illustrated by Figure 2.

### 2.4. The Generalized Divisionless Algorithm for the MT-Type Velocity Estimation Method.

**Figure 3:**
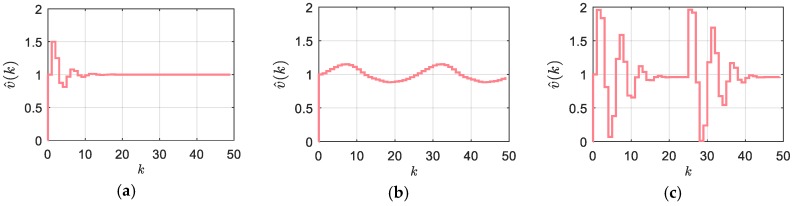
Step response of the DLMT filter: (**a**) constant *δt_k_*/*T_s_* = 0.5; (**b**) sinusoidal variation of *δt_k_*/*T_s;_* (**c**) sawtooth variation of *δt_k_*/*T_s_*.

The DivisionLess MT-type (DLMT) velocity estimation algorithm was introduced in [33]. The basic idea of the method is to calculate the velocity in fixed sampling periods, having available exact actual position readings at the sampling time instants. This would result in the average velocity within the sampling period that can be considered as the optimal velocity information. However, incremental encoders cannot provide the actual position at the sampling instants due to the asynchronous nature of the encoder pulses; the correct position evidence can be provided just for the time instant of the encoder pulse occurrence that mismatches the real position at the sampling instants. Therefore, alternative sampling instant position information should be provided. The authors in [33] propose linear extrapolation based on the read encoder position, the velocity and the measured time interval elapsed since the recent encoder pulse was acquired. However, the real velocity information, accurate in time, is not available. Instead, only the velocity estimation can be applied. In advanced motion control applications it is reasonable to assume fast sampling periods such that the velocity change in a single sampling period is close to zero. Therefore, we can use the velocity estimation from the previous sampling period. The algorithm can be described by (6) and (7):

(6) $${\hat{x}}_{k}^{a}=x_{k}+{\hat{v}}_{k-1}\delta t_{k},$$

(7) $${\hat{v}}_{k}=\frac{{\hat{x}}_{k}^{a}-{\hat{x}}_{k-1}^{a}}{T_{s}},$$

where ${\hat{x}}_{k}^{a}$ is the estimated actual position and the time instant $t_{k}=kT_{s}$, and ${\hat{v}}_{k}$ is the corresponding estimated velocity. The first equation of the algorithm (6) approximates the real position at the sampling instant, and the second Equation (7) outputs the velocity estimation, which should approximate the average velocity in the recent sampling interval. The algorithm bypasses the arithmetic division efficiently by estimating the actual position at the sampling instant. Then, the pure position difference (similar to the simple M-method) leads to the smooth velocity estimation output, which is close to the conventional MT-method velocity output. However, the algorithm is recursive, since the actual velocity estimation is defined by the previous values, as described by:

(8) $${\hat{v}}_{k}-\frac{\delta t_{k}}{T_{s}}{\hat{v}}_{k-1}+\frac{\delta t_{k-1}}{T_{s}}{\hat{v}}_{k-2}=\frac{\Delta x_{k}}{T_{s}},$$

where the input $\Delta x_{k}=x_{k}-x_{k-1}$ stands for the number of encoder pulses counted in a single sample interval. One can note that the dynamic discrete Equation (8) actually represents a linear discrete filter of a second order with time-varying coefficients, and with its right-hand side that equals the M-method (2). Due to the recursive nature of the algorithm, stability becomes an issue. It has been shown that the algorithm is asymptotically stable under the condition that the left-hand side coefficients are bounded within interval [0,1). The dynamic equilibrium ${\hat{v}}_{k}^{eq}$ of (8), given by:

(9) $${\hat{v}}_{k}^{eq}=\frac{x_{k}-x_{k-1}}{T_{s}+\delta t_{k-1}-\delta t_{k}},$$

which equals the MT-method output (3), is, therefore, attractive. It means that the DLMT algorithm output converges to the accurate velocity estimation. The step response of the filter dynamics (8) is illustrated by Figure 3 with three cases of $\delta t_{k}/T_{s}$ such that it is: (a) Constant and equals 0.5, (b) Variable by sinus waveform of two periods with the amplitude of 1 and offset of 0.5, and (c) Variable by two periods of the sawtooth waveform that varies in the interval [0,1]. The asymptotically stable response is evident in the case of (a). In the case of (b), the filter generates a sinusoidal output to provide stable convergence to the dynamic equilibrium defined by (9). In the case of (c), we can observe high velocity peaks when the sawtooth waveform generates the abrupt changes from 0 to 1 (sudden change from *δt_k_* = 0 to *δt_k_*_+1_ = *T_s_*), which are followed by the asymptotically stable convergence, although it is oscillatory. However, it should be noted that this scenario is highly unrealistic in practice.

Though the theoretical treatment of the DLMT algorithm in [33], along with the numerical cases, shows stable convergence, it does not address a scenario with blank sampling intervals, which appear at low velocity. Let encoder pulses appear in the $k_{i}$-th sampling interval, which is then followed by $n_{i}$ blank sampling intervals. If we consider that the velocity estimation is updated only in non-blank sampling intervals, and if we derive the equation of the estimated actual position at the ($k_{i}-1$)-th sampling interval as:

(10) $${\hat{x}}_{k_{i}-1}^{a}={\hat{x}}_{k_{i-1}}^{a}+{\hat{v}}_{k_{i-1}}(n_{i-1}T_{s}),$$

where ${\hat{x}}_{k_{i-1}}^{a}=x_{k_{i-1}}+{\hat{v}}_{k_{i-2}}\delta t_{k_{i-1}}$, then the output velocity estimation (as in (7)) can be derived as:

(11) $${\hat{v}}_{k_{i}}=\frac{{\hat{x}}_{k_{i}}^{a}-{\hat{x}}_{k_{i}-1}^{a}}{T_{s}}=\frac{(x_{k_{i}}-x_{k_{i-1}})+{\hat{v}}_{k_{i-1}}(\delta t_{k_{i}}-n_{i-1}T_{s})-{\hat{v}}_{k_{i-2}}\delta t_{k_{i-1}}}{T_{s}},$$

If we rearrange the upper final equation and, furthermore, simplify the notation such that we index by ${\left(.\right)}_{i}$ instead of ${\left(.\right)}_{k_{i}}$, then the discrete dynamics Equation (8) in case of some blank sampling intervals transforms into the recursive discrete equation of the second order (12) that updates in irregular intervals at $t_{i}=t_{k_{i}}=k_{i}T_{s}$, where $k_{i}=k_{i-1}+n_{i-1}+1$.

(12) $${\hat{v}}_{i}-(\frac{\delta t_{i}}{T_{s}}-n_{i-1}){\hat{v}}_{i-1}+\frac{\delta t_{i-1}}{T_{s}}{\hat{v}}_{i-2}=\frac{\Delta x_{i}}{T_{s}}$$

**Figure 4:**
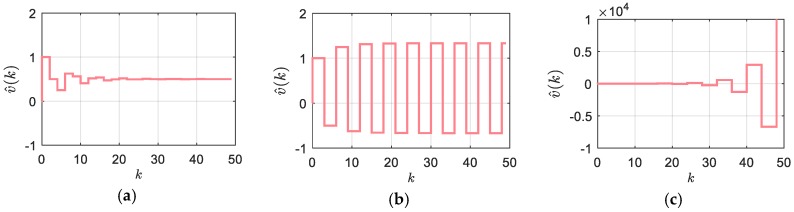
Step response of the DLMT filter in the case of blank sampling intervals (constant *δt_k_*/*T_s_* = 0.5): (**a**) *n_i_* = 1; (**b**) *n_i_* = 2; (**c**) *n_i_* = 3.

A comparison of (12) with (8) yields the difference in the second coefficient on the left-hand side of the latter equation. While the coefficients on the left-hand side of (8) are bounded by the interval [0,1) in the absolute sense, this is not the case in (12), since $n_{i-1}=\left[0|1|2|\ldots \right]$ appears in the coefficient of ${\hat{v}}_{i-1}$. It can be shown that this seriously violates the stability conditions, which are presented in [33]. Thus, the algorithms (6), (7) cannot provide asymptotically stable velocity estimation in a general case, and therefore, must be appropriately modified. Instability of the DLMT filter (12) in the case with blank sampling intervals is shown by Figure 4, with the asymptotically stable response at $n_{i}=1$ (a), the limited stability at $n_{i}=2$ (b), and the unstable response at $n_{i}=3$ (c).

In this paper, we introduce an adaptation of the DLMT algorithm to provide an asymptotically stable output in the generalized case that involves blank sampling intervals. The Generalized DivisionLess algorithm for MT-type (GDLMT) velocity estimation method is given by (13) and (14):

(13) $${\hat{x}}_{i}^{a}=x_{i}+{\hat{v}}_{i-1}\delta t_{i},$$

(14) $${\hat{v}}_{i}=\frac{{\hat{x}}_{i}^{a}-{\hat{x}}_{i-1}^{a}}{m_{i-1}T_{s}},$$

It can be shown easily that the proposed GDLMT algorithm cancels the $n_{i-1}$ term in the second coefficients of the left-hand side of (12). Thus, the dynamics equation can be rewritten such that it yields:

(15) $${\hat{v}}_{i}-\frac{\delta t_{i}}{m_{i-1}T_{s}}{\hat{v}}_{i-1}+\frac{\delta t_{i-1}}{m_{i-1}T_{s}}{\hat{v}}_{i-2}=\frac{\Delta x_{i}}{m_{i-1}T_{s}},$$

which satisfies the conditions for asymptotically stable convergence to the dynamic equilibrium:

(16) $${\hat{v}}_{i}^{eq}=\frac{1}{1-\frac{\delta t_{i}}{m_{i-1}T_{s}}+\frac{\delta t_{i-1}}{m_{i-1}T_{s}}}\frac{\Delta x_{i}}{m_{i-1}T_{s}}=\frac{x_{i}-x_{i-1}}{m_{i-1}T_{s}-\delta t_{i}+\delta t_{i-1}}.$$

**Figure 5:**
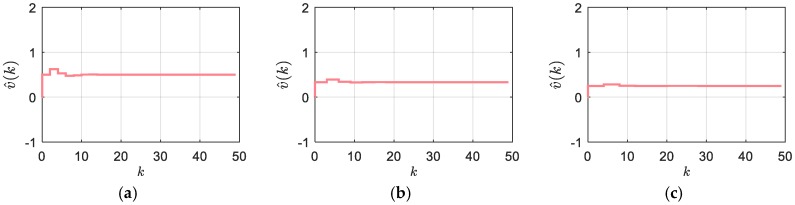
Step response of the GDLMT filter in the case of blank sampling intervals (constant *δt_k_*/*T_s_* = 0.5): (**a**) *n_i_* = 1; (**b**) *n_i_* = 2; (**c**) *n_i_* = 3.

By comparison of (16) and (4) we can conclude that the dynamic equilibrium of the GDLMT method is equivalent to the output of the MT-method. The step response of the GDLMT filter (15) is depicted by Figure 5 with the asymptotically stable response in all cases of a number of blank intervals ($n_{i}=1$ (a), $n_{i}=2$ (b), and $n_{i}=3$(c)).

One can note that the GDLMT algorithm involves the variable term $m_{i-1}T_{s}$ in the denominator of the right-hand side of the output Equation (14). It implies on-line arithmetic division, which cannot be substituted simply by multiplication with a single fixed inverse value. However, for a bounded set of $m_{i}=\left[1|2|\ldots |m_{\max}\right]$ we can calculate the inverse values off-line, since all the possible values of $m_{i}$ represent a set of constants known in advance. It is easy to calculate the inverse values of ${\left(m_{i}T_{s}\right)}^{-1}$ for a limited number of $m=1,2,\ldots ,m_{\max}$ off-line and store the results into a memory, e.g., as a zero-indexed array (during the on-line computation, the values of $n_{i}$ can be used as the index). Then the output formula (14) can be read as:

(17) $${\hat{v}}_{i}={\left(m_{i-1}T_{s}\right)}^{-1}\Delta {\hat{x}}_{i}^{a},$$

where $\Delta {\hat{x}}_{i}^{a}={\hat{x}}_{i}^{a}-{\hat{x}}_{i-1}^{a}$ and ${\left(m_{i}T_{s}\right)}^{-1}$ can be recalled from the memory during the on-line execution of the algorithm. At the bottom line, the arithmetic division involved in the GDLMT algorithm can be replaced by the arithmetic multiplication in on-line execution with real-time performance. However, note, that $m_{\max}$ determines the minimal measurable velocity. If overrun of *m* happens (such that $m>m_{\max}$), then the estimated velocity can be set to zero.

### 2.5. The Experimental System

**Figure 6:**
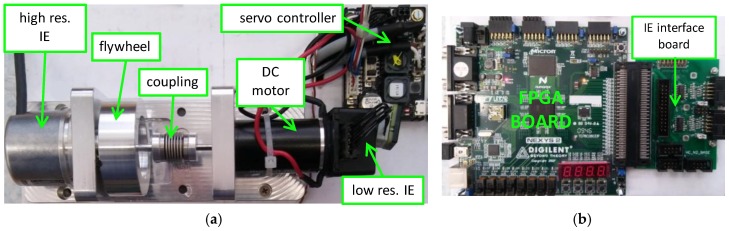

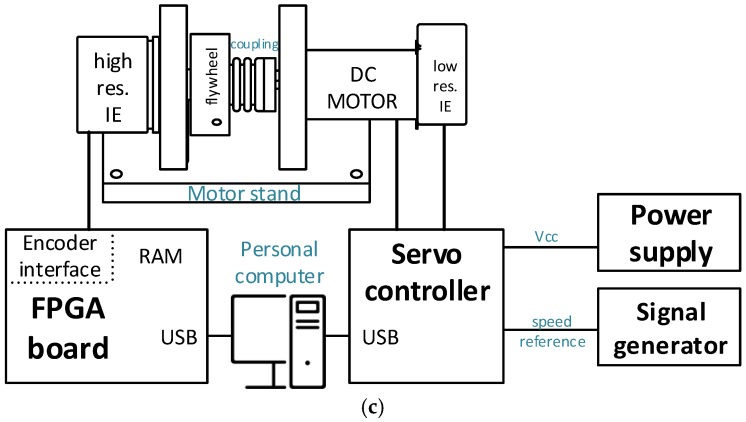
The experimental setup: (**a**) the mechanical drive system with the IE; (**b**) the processing hardware electronics with the FPGA; (**c**) the principal interconnection block sheme.

The experimental setup consists mainly of a mechanical drive system with a rotary incremental encoder (IE), and the necessary processing hardware electronics. It is shown in Figure 6. For the processing hardware, we employed a Digilent Nexys2 Board [36] with a cost optimized Xilinx Spartan XC3S1200E FPGA that is designed as a high-performance digital circuit for high-volume, consumer-oriented applications. Besides that, our board also includes lots of memory (16 MB Micron Cellular PSRAM, 16 MB Flash ROM, and additional Flash ROM for nonvolatile FPGA configurations), oscillators (50 MHz in-circuit oscillator and an additional oscillator of 125 MHz with 50 ppm in the socket for custom crystal oscillator), a high-speed Hirose FX2 expansion connector, four PMOD connectors with 8 I/O lines each, and a USB 2.0 high-speed port for the communication link to the development PC, among others. The FPGA digital circuit features numerous logic cells, distributed RAM bits, blocks of RAM, hardware multipliers with 18-bit inputs, digital clock managers, etc.

The mechanical drive system consists of a DC motor Maxon RE 25 with low-resolution incremental encoder HEDS 5540 (with 500 line counts per turn), mounted on the rear side of the motor shaft, and a high resolution Heidenhain ROD 1020 rotary incremental encoder (with 3600 line counts per turn) is linked to the front-side of the motor shaft. The latter was used for the experimental evaluation and validation of the proposed method for velocity estimation. The motor shaft and the high-resolution incremental encoder shaft are interconnected mechanically by a metal bellows coupling. Additionally, we attached a rotational flywheel on the encoder shaft in order to increase its rotational inertia, which can, consequently, reduce mechanical oscillations. The flywheel, along with the testing incremental encoder, represents the motor dynamical load. In order to damp further the mechanical disturbances from the motor side, which were demonstrated during our experimental tests by higher frequency motion oscillations, we replaced the metal coupling with a soft silicon tube in some experiments, which, on the other hand, cannot provide the proper stiffness required for highly dynamical motion. Thus, the mechanical system transformed into an elastic two-mass system and, therefore, an additional low frequency fluctuating motion occurred on the load side. The DC motor was driven electrically by a Maxon ESCON 36/2 DC Servo Controller configured in speed control mode with the low-resolution encoder in the loop. The driving signal of reference speed, connected to its analog input, was provided by the Rigol DG 1022 function generator.

The electrical lines of the high-resolution incremental encoder were connected to the FPGA board by a custom interface board on the high-speed expansion connector. The interface board contains standard RS 422 line receivers, proper analog filters and line termination. The enhanced quadrature decoder, with a clock frequency of 125 MHz, was implemented in the FPGA circuit. Thus, capture of encoder pulses and relatively accurate measurement of time intervals were performed with a resolution of 8 ns. The sampling frequency, by which we read encoder pulses and the elapsed time interval since the recent encoder pulse, was 10 kHz. The update of the velocity estimation output was performed synchronously with the sampling. The data were stored to the on-board RAM every sampling period in real-time. Thus, after an experiment was completed, the data were available for transfer in batch via the USB port to the desktop PC and stored for further off-line post processing.

**Figure 7:**
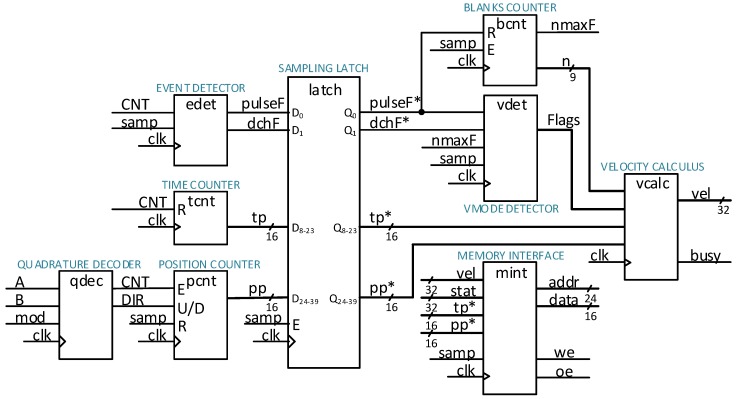
The block scheme of the velocity measurement system logic design on the FPGA.

The calculus of the velocity estimation algorithm was designed in a fixed-point format, and implemented by integer arithmetic operations. The inputs in the algorithm sampled by the period of *T_s_* = 0.1 ms are: (a) Integer number of encoder position pulses (position pulses abbreviated as pp), and (b) Integer number of clock ticks counted from the recent encoder pulse (time pulses abbreviated as tp). The longest measurable time interval in the current implementation is limited by the 16-bit register and the clock frequency of 125 MHz, that yield around 0.5 ms. Additionally, we can count up to 511 blank sampling periods. The velocity output is provided in a 32-bit signed Q-format with 18 bits for the integer part (including the sign bit) and 14 bits for the fractional part. It is given in units of the number of encoder position pulses per sampling period (pp/*T_s_*). The algorithm was coded in VHDL. The calculation of the algorithm was organized in sequential steps such that it performs in only a few clock cycles. For the multiplication arithmetic operations, we employed the hardware multipliers embedded in the FPGA, while the addition and subtraction operations were performed by the general FPGA logic cells. Normally, the calculus is performed every sampling period if at least a single encoder pulse occurs. Otherwise, the velocity is not updated and an old value is preserved at the output. If the count of blank sampling periods exceeds a maximum number, or in a case of detected direction change, then the output is forced to zero. The calculus of the velocity can be re-enabled when two sampling periods with encoder pulses in the same direction appear. The principal core framework logic circuit design implemented on the FPGA, is shown by Figure 7.

All the modules featured by the block scheme operate synchronously by the basic clock signal ‘clk’, and ‘samp’ is the trigger signal generated at the sampling instants in the duration of a single clock cycle. The modules left from the ‘latch’ represent the capturing section with the encoder signals as the inputs ‘A’ and ‘B’, whereas the modules on the right side process the signals sampled by the ‘latch’. The sampled signals (and the processed signals as well) are logged promptly to RAM. The result of the processing section is the estimated velocity ‘vel’. The ‘QUADRATURE DECODER’ reads encoder signals every clock cycle, and generates ‘CNT’, which reflects a position pulse to be counted, and ‘DIR’ that determines the counting direction (incrementing or decrementing). We can select two modes of decoding, i.e., in the ‘X1’ mode, the number of position pulses generated on the decoder output in one revolution equals the encoder line counts, whereas in the mode ‘X4’ the number of the position pulses are quadruple. The ‘POSITION COUNTER’ then counts the position pulses. The ‘TIME COUNTER’ measures the elapsed time interval since the recent position pulse by counting the clock cycles. The ‘EVENT DETECTOR’ module generates the event flags for “motion direction change” (the ‘dchF’ output) and for “position pulse occurred” (the ‘pulseF’ output) in a recent sampling period. The output data from the above-mentioned modules are captured by the ‘latch’ module at the sampling instants. The latched signals are denoted with a supplemented asterisk (*). The ‘BLANKS COUNTER’ counts blank sampling periods (a contiguous series of sampling periods with no position pulse occurrence), which is provided at the ‘n’ output. The ‘nmaxF’ is the output flag, which signals that the ‘n’ has reached a maximum value. The latched pulse flag ‘pulseF*’ reset the value of the counted blanks. Then the ‘VMODE DETECTOR’ module generates velocity mode flags that are required for the control logic of the arithmetic module ‘VELOCITY CALCULUS’. The latter performs calculation of the velocity estimation using counted position pulses (‘pp*’), counted time pulses (‘tp*’), and counted blank sampling periods (‘n*’). The ‘busy’ output flag indicates the duration of the calculus. The sequence of positive integers’ reciprocals (1/1, 1/2, 1/3, …, 1/511) was calculated off-line, and organized as a look-up table in the embedded block RAM. The size of the look-up table limits the maximum number of counted blank periods.

**Figure 8:**
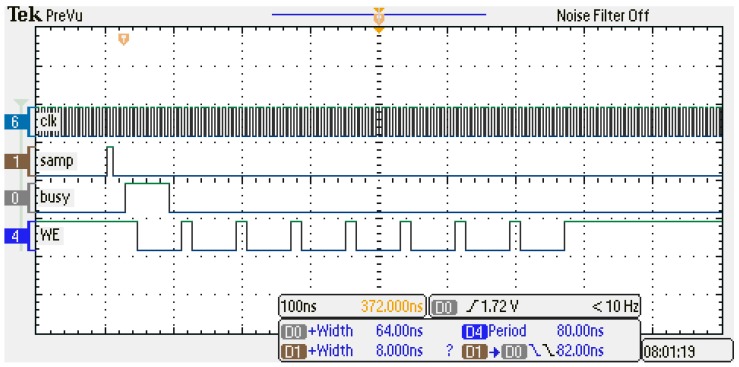
The timing diagram of the core FPGA circuit.

The timing diagram, which includes the interval required for the calculus and writing the data to the RAM (by the ‘MEMORY INTERFACE’ module), is shown by Figure 8. The logic signals from the figure were captured by a Tektronix MSO2014 oscilloscope (1 Gsps). The velocity estimation algorithm starts soon after the fresh readings are available for the processing. It provides the result in 64 ns. Thus, the output is available in less than 100 ns since the positive edge of the sampling instant (the ‘samp’ trigger pulse). Writing to the RAM, which is enabled by the low level of the write-enable (‘we’) signal, starts already during the computation phase. It transfers the data word-by-word in the following sequence of 8 words: a single word of the ‘pp*’ data, a single word of the ‘tp*’ data, two reserved words, a double word of ‘stat’ data, and a double word of ‘vel’ data.

The consumption of the FPGA resources for the experimental design solution presented above is relatively small. For the calculation process, we used 5 dedicated 18 × 18 hardware multipliers, two blocks of RAM, and the total number of occupied logic cells was less than 5%.

## 3. Experimental Results

We performed various experiments in a wide speed range in order to test and validate the velocity measurement by the proposed GDLMT algorithm implemented on the FPGA board. The experiments covered the range from high speed to low speed. We consider high speed in the case of a large number of encoder pulses per sampling period, e.g., 50 pulses and more, and low speed is about 1 pulse per sampling period or below. In order to achieve high-speed motion, we employed the motorized drive by a ramp or a trapezoidal speed profile with a constant acceleration/deceleration phase, respectively. The motor shaft and the encoder shaft with the flywheel were linked by a metal bellows coupling. However, due to considerable system disturbance from the motor side that appears as undesired high-frequency oscillations in motion, we replaced the stiff coupling with a soft silicon tube in some experiments. Though it resulted in smoother motion, low-frequency fluctuations appeared due to the soft coupling in the two-mass system with the motor rotor on one side and the flywheel on the other side. In spite of the disturbing motion oscillations and fluctuations, the velocity estimation could provide proper velocity information in all circumstances. Nevertheless, we performed low-speed experiments driving the encoder only by hand, i.e., manually, or by a gravitational load, in order to reduce disturbances even further and to achieve a smooth motion.

**Figure 9:**
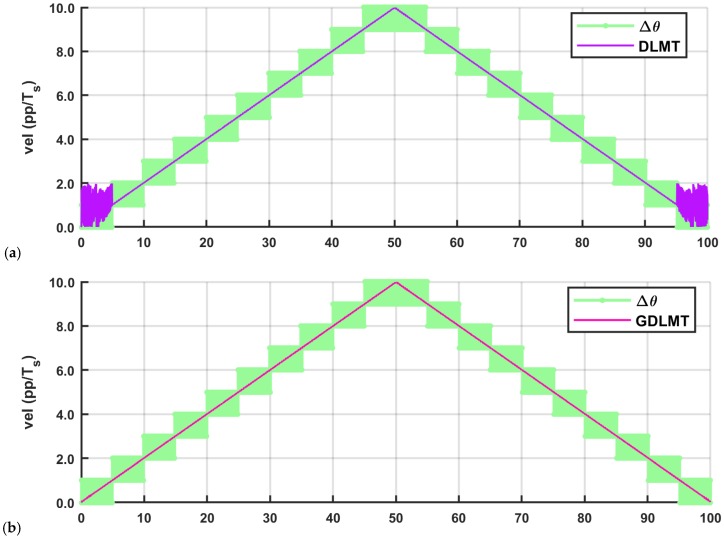
Comparison of the DLMT method and the GDLMT method at ramp speed: (**a**) pure DLMT method; (**b**) GDLMT method.

Figure 9 depicts the experimental results with a ramp speed profile (motion oscillations are not shown due to the relatively large scale). We compared the performance of the pure DLMT algorithm, and the proposed GDLMT algorithm with the adaptation for stable performance at low-speed. The GDLMT algorithm was computed on-line on the FPGA platform by integer arithmetic operations with data numbers in an appropriate Q-format. The DLMT velocity was computed on the desktop computer for off-line post-processing by single floating-point arithmetic. In both cases, the input data were the same. The velocity results are given in “position pulses per sampling period” (pp/*T_s_*). Δ*θ* denotes the integer number of encoder pulses read in a single sampling period, i.e., the simple M-method (the light green curve trace). It is characterized by stepping and alternating, as expected. In both diagrams of Figure 9, the MT-type velocity estimations show smooth performance in the whole speed range except at low speed below 1 pp/*T_s_*. In this range, the DLMT method (the purple curve trace) demonstrated highly oscillating behavior with an erroneous result, whereas the proposed GDLMT algorithm (the pink curve trace) shows stable performance clearly, similar as in the speed range beyond 1 pp/*T_s_*.

**Figure 10:**
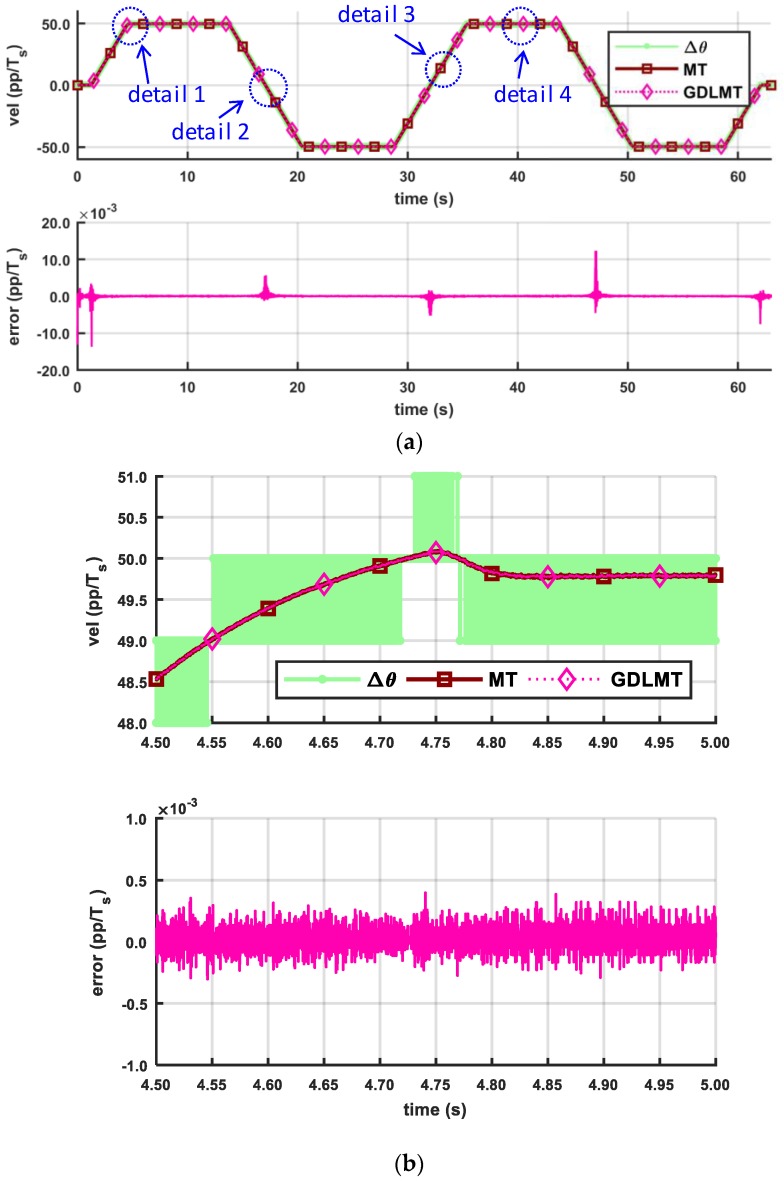

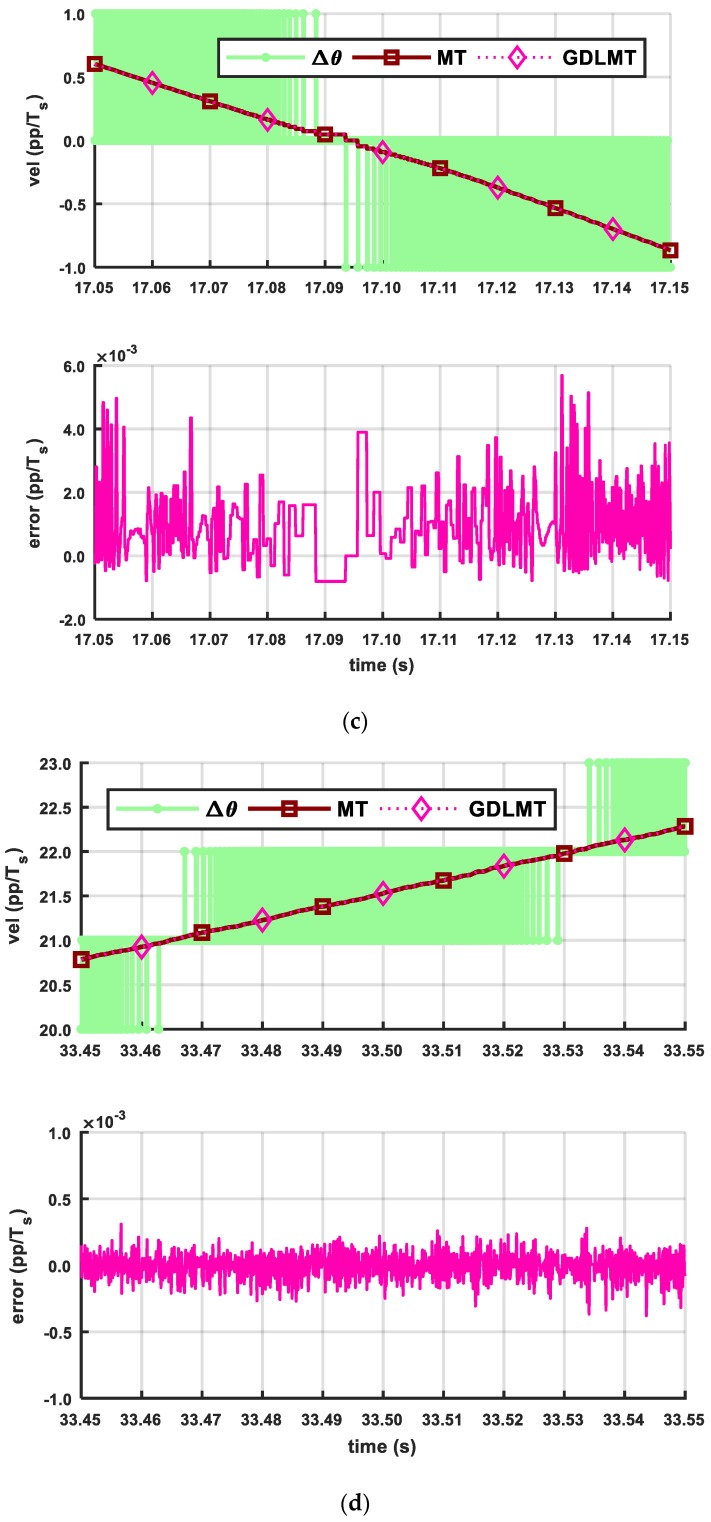

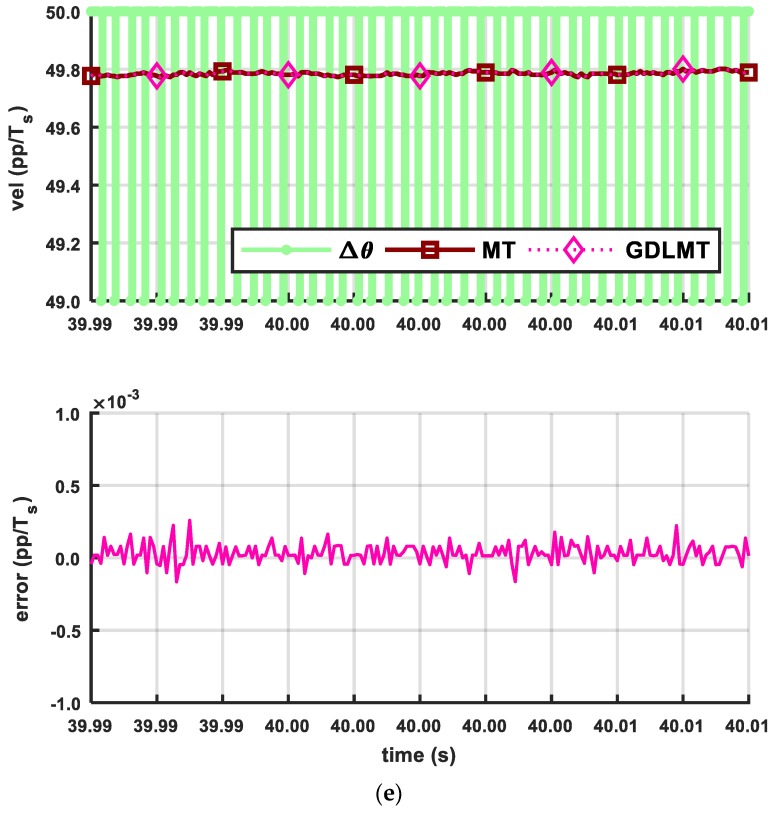
Comparison of the MT method and the GDLMT method at high speed: (**a**) full time interval; (**b**) detail 1 with velocity overshoot (peak velocity); (**c**) detail 2 with velocity zero crossing; (**d**) detail 3 with ramp velocity (constant acceleration), and (**e**) detail 4 with constant velocity.

**Figure 11:**
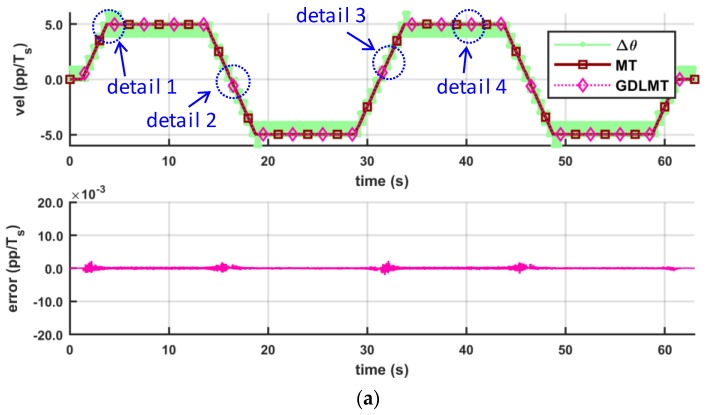

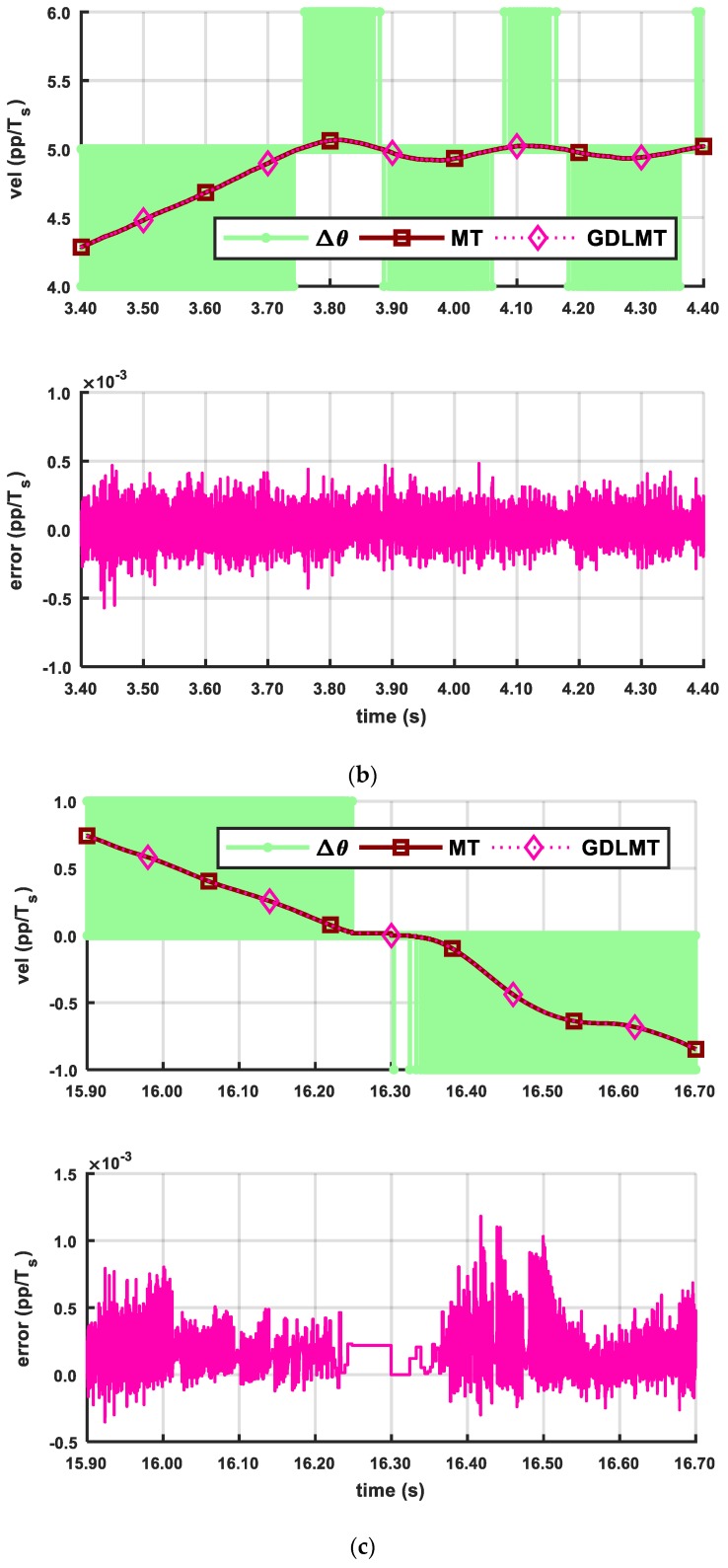

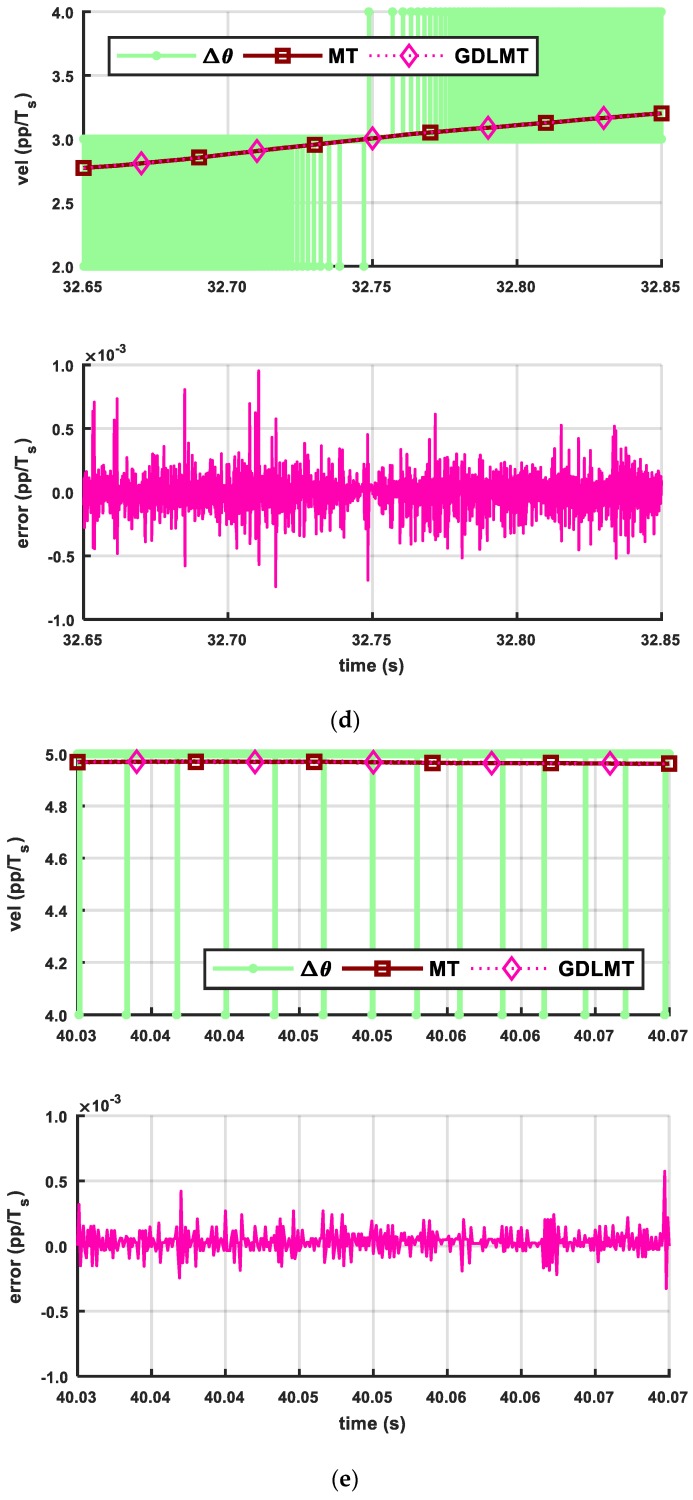
Comparison of the MT method and the GDLMT method at medium speed: (**a**) full time interval; (**b**) detail 1 with velocity overshoot (peak velocity); (**c**) detail 2 with velocity zero crossing; (**d**) detail 3 with ramp velocity (constant acceleration), and (**e**) detail 4 with constant velocity.

**Figure 12:**
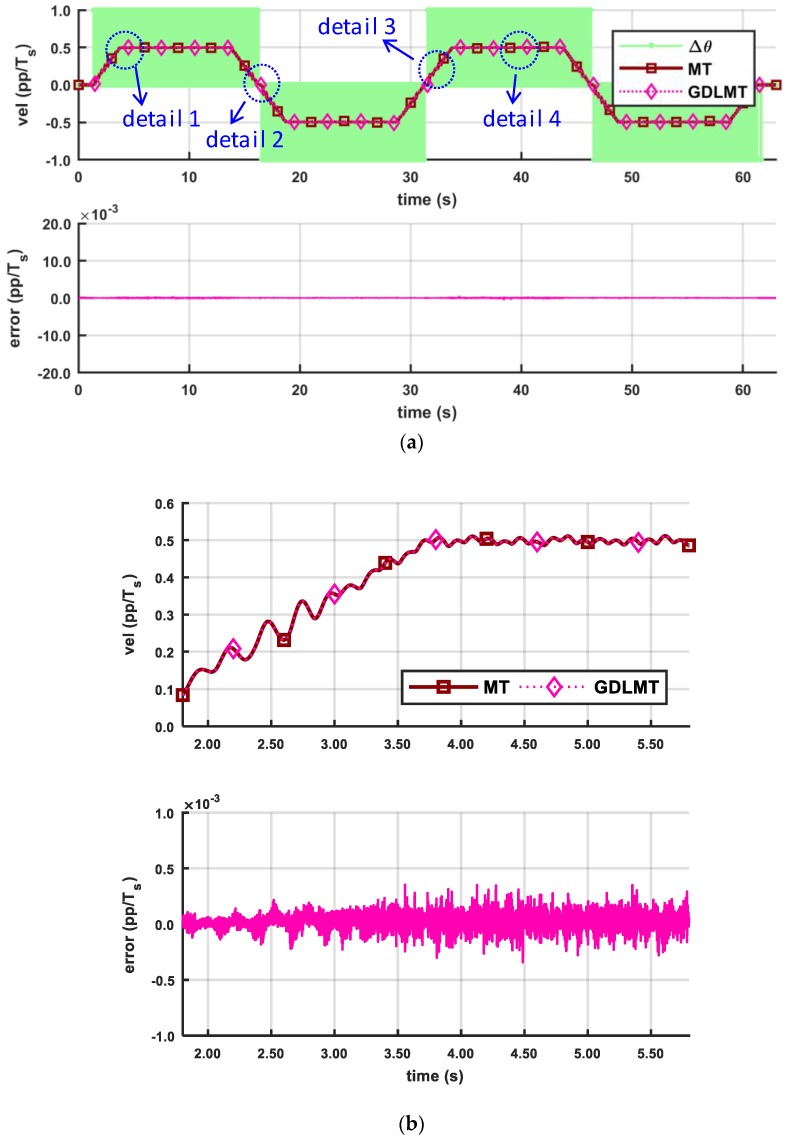

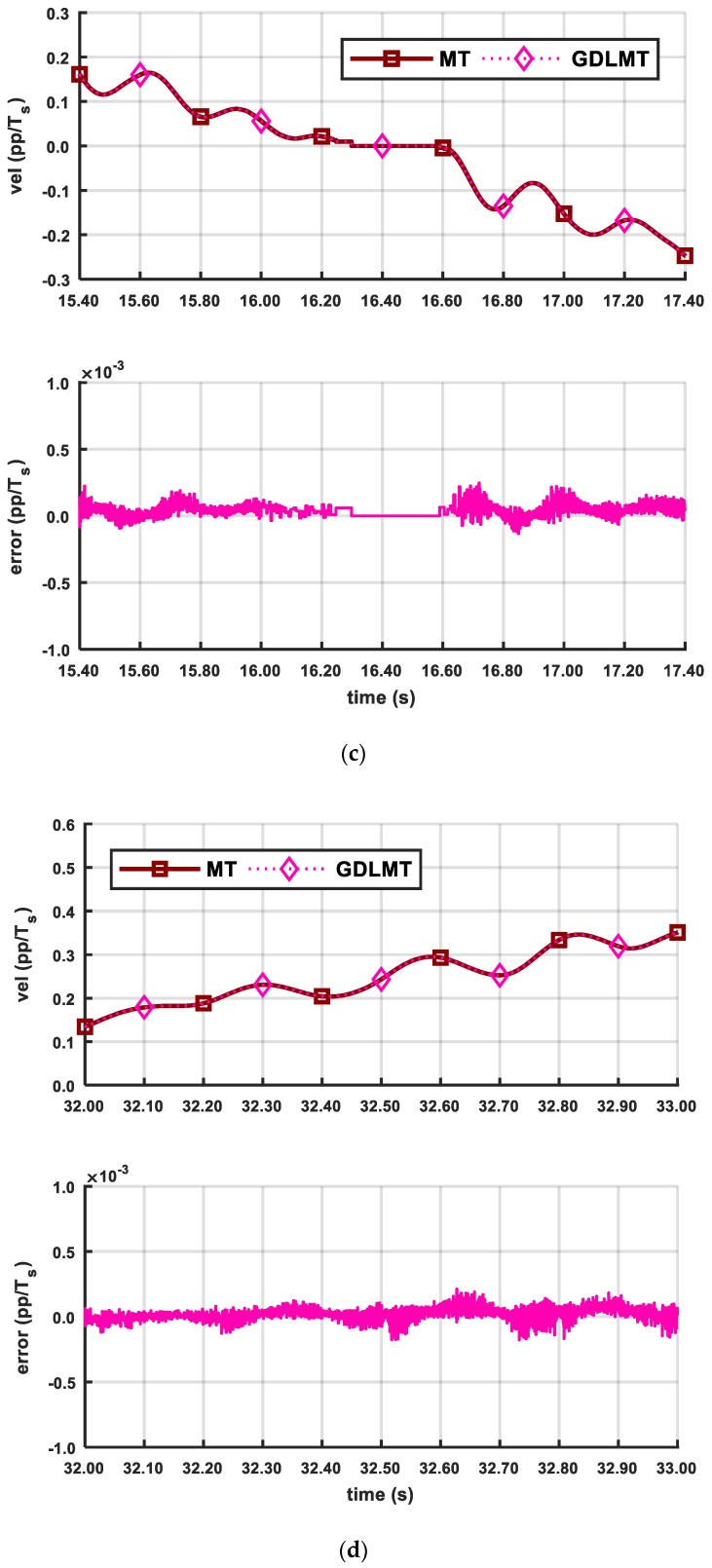

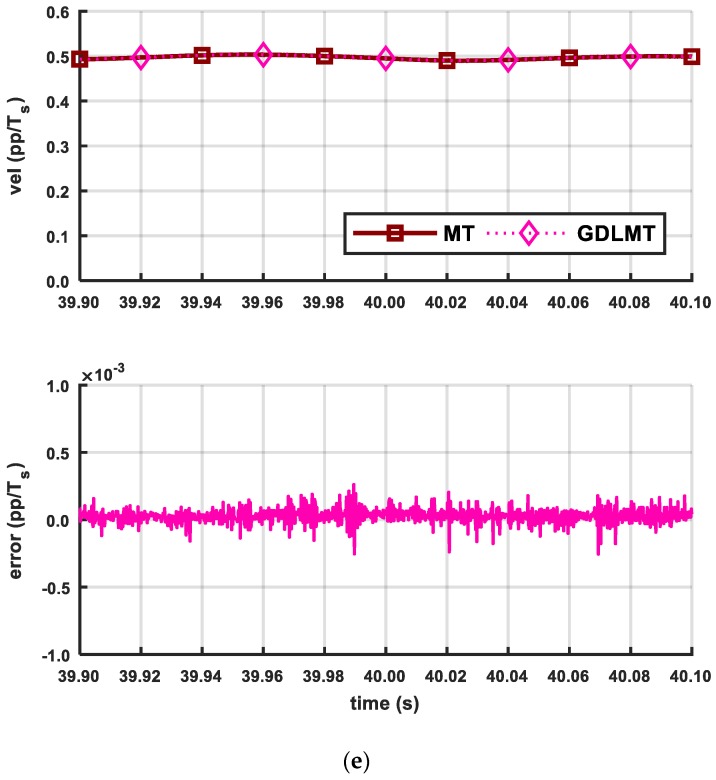
Comparison of the MT method and the GDLMT method at low speed: (**a**) full time interval; (**b**) detail 1 with velocity overshoot (peak velocity); (**c**) detail 2 with velocity zero crossing; (**d**) detail 3 with ramp velocity (constant acceleration), and (**e**) detail 4 with constant velocity.

The next three figures show the experimental results obtained by a trapezoidal profile at high speed of 50 pp/*T_s_* (with the stiff metal bellows coupling, Figure 10, at medium speed of 5 pp/*T_s_* (with the soft silicon coupling, Figure 11, and at low-speed of 0.5 pp/*T_s_* (with the soft silicon coupling, Figure 12, respectively. With these figures we provide mainly a comparison with the conventional MT-method (the dark red curve trace), which was computed on the desktop computer for off-line post-processing with the same input data as in the case of the GDLMT algorithm (the pink curve trace) implemented on the FPGA platform. The upper diagrams show the velocity traces including the simple M-method (the light green curve trace), while the bottom diagrams provide the velocity estimation error, which is computed such that the MT-method is considered as a reference (i.e., the difference between the MT-velocity trace and the GDLMT-velocity trace). On every Figure we added some characteristic details in zoom: (i) Detail 1 with a velocity overshoot (peak velocity), (ii) Detail 2 with a velocity zero crossing, (iii) Detail 3 with a ramp velocity at constant acceleration, and (iv) Detail 4 with a constant velocity. Figure 10 shows the results at high speed. The estimation error was below 0.001 pp/*T_s_*, except around the velocity reversals, with error peaks that could exceed 0.01 pp/*T_s_*. The lowest error was in the case of the constant velocity (Figure 10e). Figure 11 shows the results at medium speed. The estimation error was very low, not exceeding only a few ten-thousandths pp/*T_s_* during the whole experiment interval. Within the selected details, the error stayed below 0.001 pp/*T_s_* practically in all cases (with a slight exception in the case of zero crossing). The lowest error was again in the case of the constant velocity (Figure 11e). In the low speed experiment (Figure 12), the error remained even below 0.0005 pp/*T_s_*. However, the velocity traces show slowly fluctuating motion that arose due to the soft shaft coupling. 

The velocity curve traces of the MT-method and of the GDLMT-method practically overlap in all cases, thus, relatively high estimation accuracy was preserved. Nevertheless, the stability of the GDLMT algorithm performance was not jeopardized in any case. 

**Figure 13:**
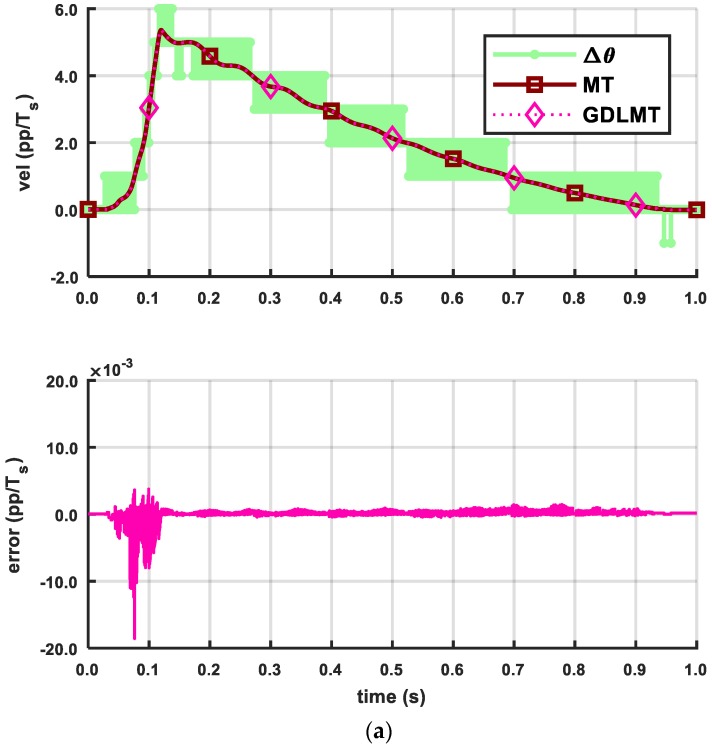

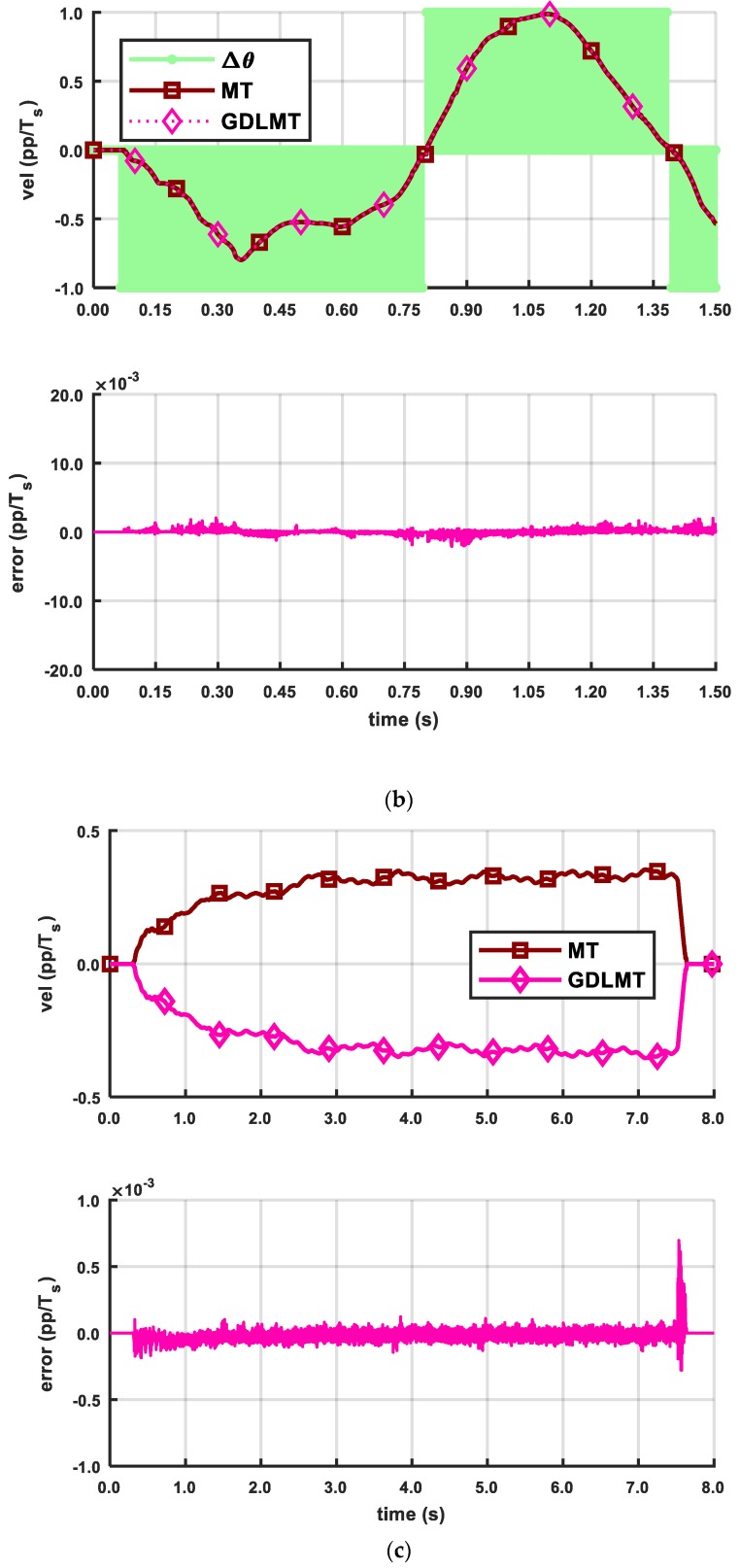

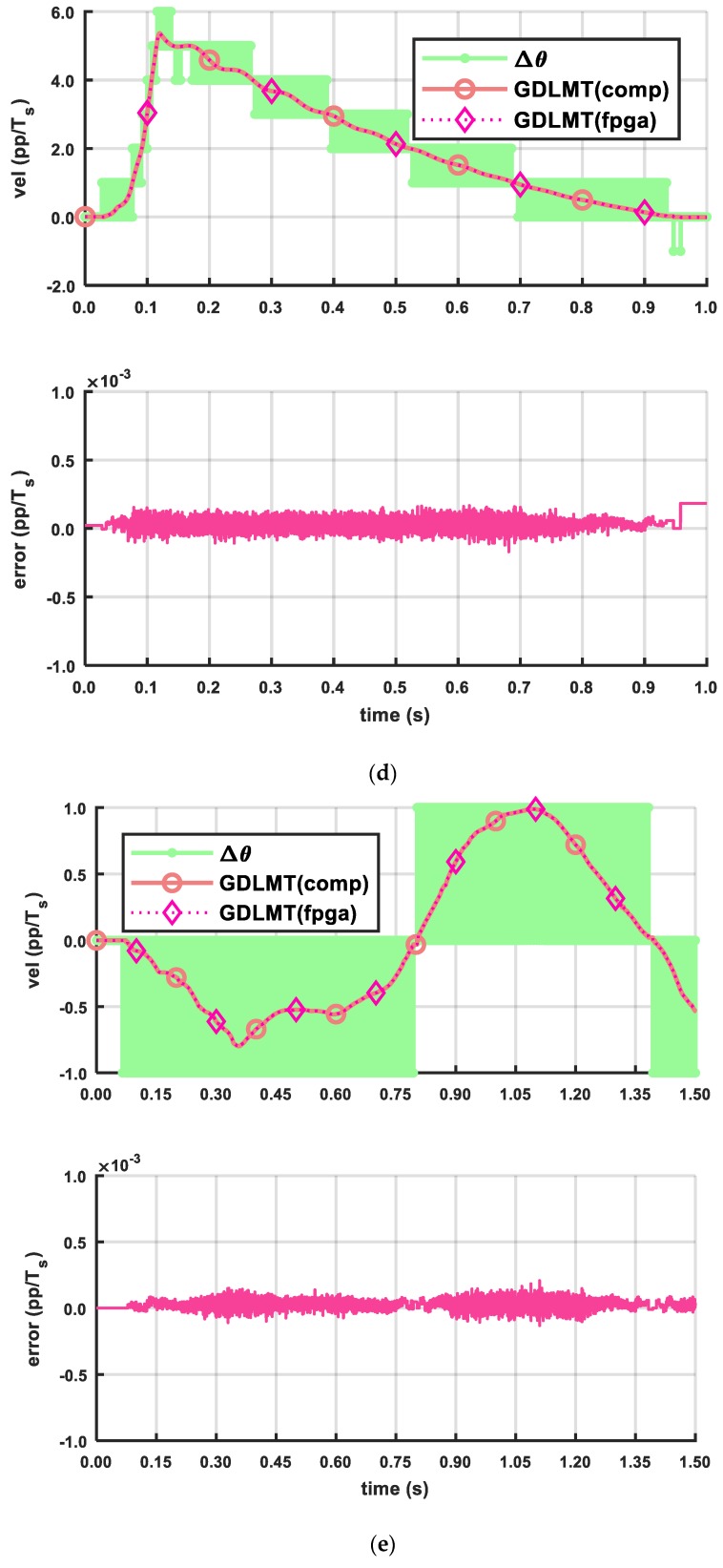

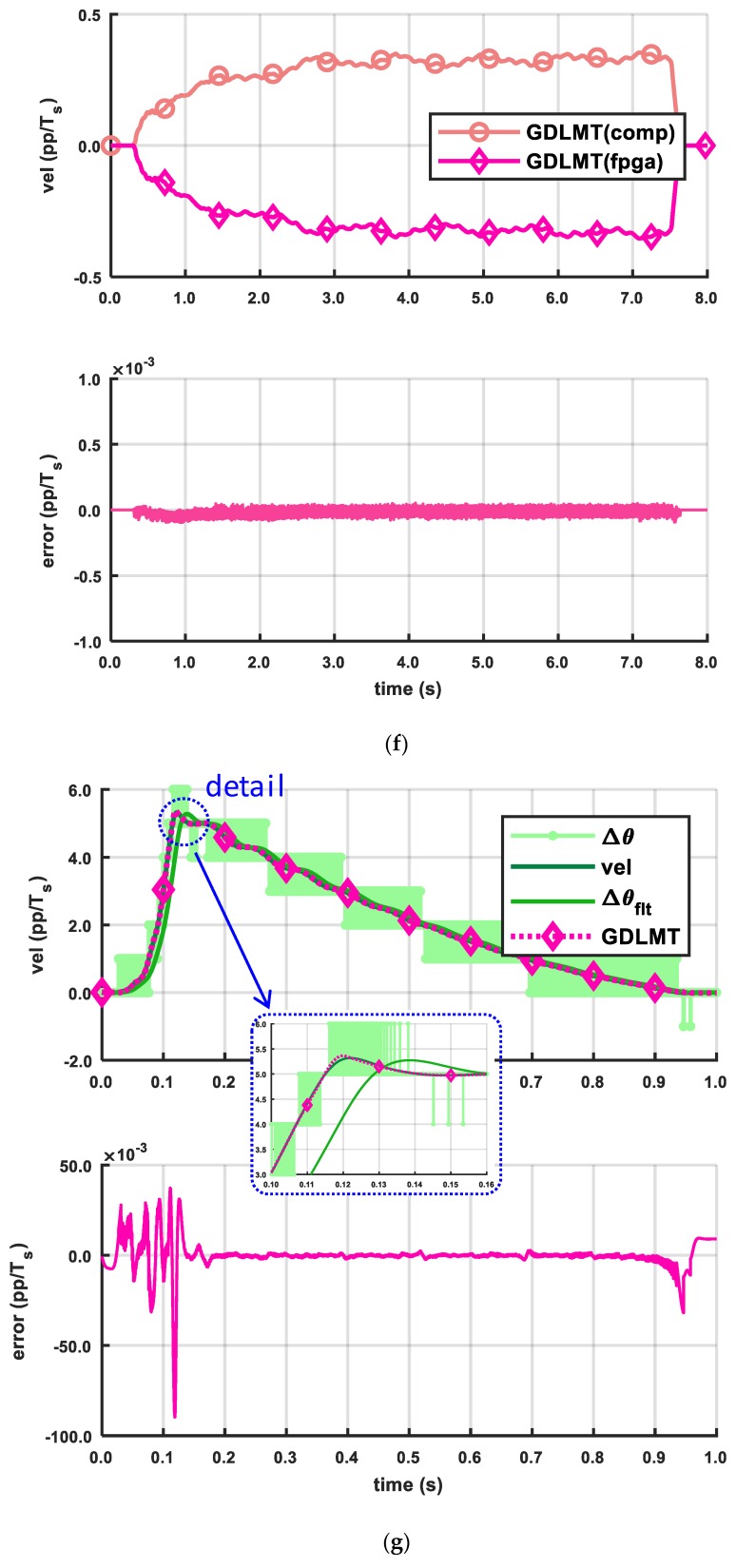

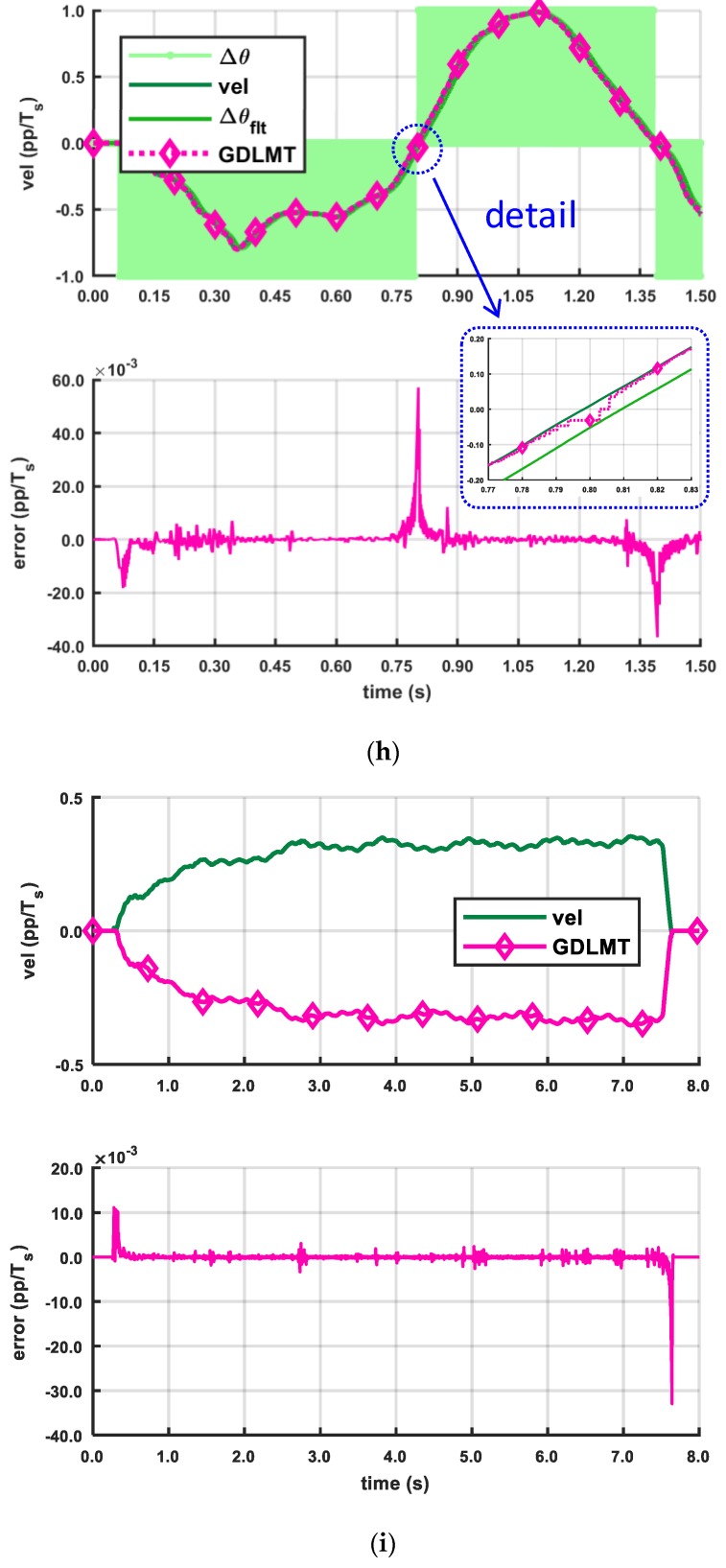
Experiments with non-motorized encoder driving at medium and low speed: (**a**,**d**,**g**) abrupt manual start up followed by self-slowdown; (**b**,**e**,**h**) manual sinusoidal-like motion; (**c**,**f**,**i**) driving by gravitational load at near-constant very low speed.

Further experiments were performed driving the encoder shaft with the flywheel manually or by gravitational load. Thus, we removed the main source of the disturbance and achieved highly smooth motion. The results shown by Figure 13 are organized by a 3 × 3 matrix. In the three columns we show the experiments as follows: (i) The first column shows the experiment with abrupt manual start up to the medium speed, which is then followed by self-slowdown at moderate deceleration (Figure 13a,d,g); (ii) The second column shows the experiment with manual driving of the encoder shaft obtaining sinusoidal-like motion at low speed (Figure 13b,e,h); and (iii), The third column shows the near-constant very low speed experiment with a vertically hanging gravitational load on a string, which linked the load over the pulley to the encoder shaft (Figure 13c,f,i). In the three rows, we show: (i) The comparison of the GDLMT algorithm (computed on-line on the FPGA platform) with the conventional MT-method (computed off-line on the desktop computer); (ii) The comparison of the FPGA implementation of the GDLMT algorithm (GDLMT(fpga)) with the GDLMT-method computed in a single floating-point format off-line on the desktop computer (GDLMT(comp)); and (iii) Comparison with the so-called actual velocity obtained by a non-causal discrete zero-phase low-pass filter (similar to the approach presented in [12]), and the velocity estimation, which is normally provided in conventional motion control applications by real-time low-pass filtering of encoder pulses read in a single sampling period (filtering the M-method). The upper diagrams show velocity traces, whereas the bottom diagrams feature error traces. In the case of comparison (iii), the filtered traces were obtained by off-line post-processing the stored data on the desktop computer, and both filters performed on the recorded data of the M-type velocity were designed by Butterworth low-pass filter of 2nd order: (a) The non-causal zero-phase filter with the cut-off frequency of 50 Hz, and (b) The causal phase-lag filter with the cut-off frequency of 20 Hz, respectively. The error traces were computed: (i) in the first row as the difference between the MT-velocity trace and the GDLMT-velocity trace; (ii) in the second row as the difference between the GDLMT (comp)-velocity trace and the GDLMT (fpga)-velocity trace); and (iii) in the third row as the difference between the so called actual velocity trace and the GDLMT-velocity trace.

In all the experimental results which are depicted by Figure 13a–c in the first row, the MT-velocity (dark red curve trace) and the GDLMT-velocity (pink curve trace) practically overlap (thus, in the third column experiment with the near-constant low speed, the GDLMT velocity trace is plotted by an inverse sign in order to provide better transparency of the plots). We can observe extremely low velocity estimation error, which increased only in the case of high acceleration in Figure 13a or in the case of high deceleration in Figure 13c.

In the second row of Figure 13 we can observe a comparison of the FPGA-computed GDLMT-velocity (pink curve trace) with the GDLMT-velocity computed off-line in a single floating-pointing format (light red curve trace). The error traces were computed off-line as well, such that the computer GDLMT-velocity was considered as a reference. Again, the velocity traces practically overlap (as shown by Figure 13d–f, and the error is kept close to zero (below 0.0005 pp/*T_s_*) in all the experiments.

In the last row, (Figure 13g,h,i), we compare the GDLMT-velocity (pink trace curve) with the so-called actual velocity (denoted as “vel”, and plotted with a dark green curve trace) and the filtered M-velocity (denoted as “Δ*θ*_flt_”, and plotted with a normal green curve trace). We also provide the error computed by the so-called actual velocity as a reference used in the comparison with the on-line computed GDLMT velocity.

The actual velocity and the GDLMT-velocity practically overlap, however, at high acceleration, deceleration and the zero crossings, the error increased (see the details in Figure 13g,h). The main causes for the increased error are related to the process of generating the so-called actual velocity trace, i.e., (a) The low-pass filtering nature, and (b) Disregarding the timing information in the occurrence of the encoder pulses. Furthermore, the zero-crossing error is inherent to the conventional MT-type estimation approach, which produces output updates only in the case of position pulse occurrence within a recent sampling period, i.e., without new position pulses the algorithm has no new information to perform the estimation. In order to deal with this problem, a special approach is required, which is out of the scope of this paper. However, on the other hand, we can observe a considerable phase-lag in the case of the causal low-pass filter, which presents a common real-time solution in motion control applications.

## 4. Discussion

The experimental results of the GDLMT-algorithm for velocity estimation by an incremental encoder show stable performance and a very high degree of matching with the conventional MT-method at various operating conditions. Nevertheless, the stability of the GDLMT algorithm performance was not jeopardized in any case, moreover, the asymptotical convergence was also clearly shown in the experiments. A typical error was below 0.1/100 (pp/*T_s_*), which is extremely low. However, good accuracy of the time interval measurement and high sampling rate are very important in order to keep the error low. Though at high acceleration and at velocity reversals the error peaks were significantly larger, i.e., 1/100 (pp/*T_s_*), the velocity estimation method performed well in a wide speed range with a motor driven encoder shaft, as well as in the case of a manually driven encoder shaft. In the latter case, we could achieve highly smooth motion, while, in the motorized case, the motion was fluctuating and oscillating. However, it did not affect the method performance in any way. Hence, the results validated the proposed algorithm.

The proposed algorithm is simple by purpose, since it is meant for implementation on a cost optimized FPGA platform. Hence, the required resources for the implementation are an issue. The lowest detected velocity in an MT-type velocity estimation method is related to the longest time interval that can be measured by the hardware. However, it is linked with the counter of blank sampling periods that comes into effect in the case of widely spread encoder pulses. The counter itself is not a problem in the FPGA implementation; however, the method requires multiplication with the inverse of the counted blanks in order to provide stable behavior. Since the values are calculated in advance off-line and stored into the FPGA memory, the maximum length of a contiguous blank interval determines the size of the required memory. Thus, the minimum detected velocity and required FPGA resources are a design trade-off, though it is not a problematic issue.

As shown in the paper, we have proven that the algorithm can be implemented efficiently on the FPGA-based platform and provide accurate velocity estimation simultaneously (though the presented experimental setup is not fully optimized yet). It should be noted that the applied experimental FPGA-platform offers overwhelming resources which are not all required to such an extent, however, they make the experimental work easier. However, further optimization in terms of the FPGA implementation is possible towards smart incremental encoders with a position and a smooth velocity output.

## 5. Conclusions

The paper deals with FPGA implementation of the MT method for velocity estimation by an incremental encoder. The conventional method involves arithmetic division, which presents a serious obstacle for implementation in a digital circuit such as an FPGA. Thus, we modified the method and introduced an algorithm which bypasses the arithmetic division efficiently. The whole algorithm can be implemented by simpler arithmetic operations such as multiplication, addition and subtraction only. We demonstrated the proof of the concept, i.e., that the implementation of the proposed divisionless MT algorithm on a real cost optimized FPGA is possible. In our experimental FPGA configuration the resources consumption is minimal and the result is available in only a few clock cycles, yet providing similar velocity estimation performance as the conventional MT-method. Thus, in the future, the proposed solution can be supported on a small size low-cost FPGA (with a cost of only few dollars), which offers only moderate resources and thus cannot provide a high performance computation engine required for complex algorithms of the arithmetical division in real-time processing of advanced motion control applications.
